# Exposure to Cigarette Smoke Augments Post-ischemic Brain Injury and Inflammation via Mobilization of Neutrophils and Monocytes

**DOI:** 10.3389/fimmu.2019.02576

**Published:** 2019-11-07

**Authors:** Handong Li, Xiuping Li, Siman Gao, Dan Wang, Xiaolin Gao, Yujing Li, Xuejiao Wang, Zhigang Cui, Hongshan Ma, Qiang Liu, Minshu Li

**Affiliations:** ^1^Department of Neurology, Tianjin Neurological Institute, Tianjin Medical University General Hospital, Tianjin, China; ^2^Center for Neurological Diseases, The Third People's Hospital of Datong, Datong, China; ^3^Barrow Neurological Institute, St. Joseph's Hospital and Medical Center, Phoenix, AZ, United States; ^4^China National Clinical Research Center for Neurological Diseases, Beijing Tiantan Hospital, Capital Medical University, Beijing, China; ^5^Advanced Innovation Center for Human Brain Protection, Capital Medical University, Beijing, China; ^6^Beijing Key Laboratory of Translational Medicine for Cerebrovascular Disease, Beijing, China

**Keywords:** stroke, cigarette smoke, inflammation, immune response, inflammasome

## Abstract

Cigarette smoke is a major preventable risk factor of ischemic stroke. Cigarette smoke induces a significant increase in circulating leukocytes. However, it remains unclear to what extent and by what mechanisms smoke priming influences stroke severity. Here we report that exposure to cigarette smoke exacerbated ischemic brain injury in mice subjected to transient middle cerebral artery occlusion (MCAO). The augmentation of neurodeficits and brain infarction was accompanied by increased production of pro-inflammatory factors and brain infiltration of neutrophils and monocytes. Prior to brain ischemia, exposure to cigarette smoke induced mobilization of peripheral neutrophils, and monocytes. Furthermore, the detrimental effects of smoke priming on ischemic brain injury were abolished either by pharmacological inhibition of the recruitment of neutrophils and monocytes or by blockade of the NLRP3 inflammasome, an effector protein of neutrophils and monocytes. Our findings suggest that cigarette smoke-induced mobilization of peripheral neutrophils and monocytes augments ischemic brain injury.

## Introduction

Despite progresses in reperfusion therapies, ischemic stroke remains a major cause of death and disability worldwide. Compiling evidence has demonstrated that inflammatory responses to cerebral ischemia plays an important role in various stages of stroke pathobiology and outcome. Initiated by the cessation of blood flow, the activation of neuroglia, recruitment of peripheral leukocytes, and release of proinflammatory factors from the ischemic region together contribute to post-ischemic brain injury (1, 2).

Tobacco use is a major preventable risk factor for ischemic stroke. Reportedly, smokers have a two- to four-fold increased risk of stroke than non-smokers (3). Cigarette smoking also has a strong link to stroke severity, disability, and length of inpatient stay (4). A significant increase in circulating leukocytes, such as neutrophils and monocytes, and pro-inflammatory factors, such as C-reactive protein and interleukin (IL)-6, has been observed in smokers (5–8), suggesting that CS induces priming of peripheral immune cells. However, still unclear is to what extent and by what mechanism smoke priming influences the pathological responses to brain ischemia.

Although previous studies in experimental models have reported a possible link—that nicotine, a major component of cigarette smoke, influences post-ischemic brain injury (9, 10)—the extrapolation of these findings toward the *in vivo* scenario is not straightforward because smoke inhalation contains more than 4,000 other components (11). To address this question, we investigated the effects of cigarette smoke on post-ischemic brain injury and inflammation, and we determined the contributions of cigarette smoke-induced priming of peripheral immune cells to the effects of cigarette smoke in mice subjected to transient middle cerebral artery occlusion (MCAO).

## Materials and Methods

### Animals

All animal experiments were approved by the Institutional Animal Care and Use Committees of Tianjin Neurological Institute (Tianjin, China). This study was conducted in accordance with the National Institutes of Health Guide for the Care and Use of Laboratory animal in China. Male C57BL/6 mice (7–8 weeks old, 20–25 g body weight) were purchased from the Vital River Laboratories (Beijing, China). Animals were housed in pathogen-free conditions at the animal facilities under a standardized light-dark cycle, and they were provided with free access to food and water. All animal experiments were designed, performed, and reported according to the Animal Research: Reporting of *in Vivo* Experiments guidelines. Animals were randomly assigned to experimental group.

### Cigarette Smoke Exposure Protocol

Mice were exposed to cigarette smoke using a whole-body smoke exposure system (Yuyan Instruments Co., Ltd. Shanghai, China). Mice were exposed to 12 cigarettes (CHIENMEN, Peking, China) for a period of ~50 min twice a day for 4 days. Animals receiving exposure to normal air were used as controls as previously described (12).

### Analysis of Total Particulate Matter in Exposure Chamber

To determine the concentrations of total particulate matter (TPM) in the smoke exposure box, samples were collected on filters provided by Yuyan Instruments (Shanghai, China) at a collection rate of ~10 L/min for 5 min. TPM concentrations were calculated based on the mass collected on the filters and the total volume of air drawn through the filter (12).

### Cotinine, Artery Blood Gas, and Cerebral Blood Flow Measurement

Cotinine levels were measured by ELISA (Bio-Quant, San Diego, CA) in serum obtained by incubating whole blood isolated from animals within 1 h after exposure for 30 min at 37.8°C, followed by centrifugation. Artery blood gas was measured by the Cobas B 123 POC System Blood Gas Analyzer (Roche, Mannheim, Germany). Approximately 100 μl blood sample with anticoagulant was obtained from mice carotid arteries under isoflurane-induced anesthesia. Mice cerebral blood flow (CBF) was measured by a PeriCam PSI laser speckle contrast imager (Perimed AB, Stockholm, Sweden). After 4 days' smoking, mice were anesthetized by isoflurane on day 5, the mouse heads were immobilized, and a midline scalp incision was made for imaging. The incision was sutured by 5-0 surgery silk thread after 1 min imaging. Body temperature was recorded and maintained at 37°C using an electric warming blanket during the operation.

### Middle Cerebral Artery Occlusion (MCAO) Model

A transient MCAO model was induced by 60 min focal cerebral ischemia and reperfusion using a filament method, as previously described (13, 14). Briefly, mice were anesthetized by inhalation of 3.5% isoflurane and maintained by inhalation of 1.0–2.0% isoflurane in 70% N_2_O and 30% O_2_ using a face mask. A 6-0 nylon filament with a rounded tip was inserted into the right MCA to occlusion for 60 min. Reperfusion was established when the filament was withdrawn back to the common carotid artery. Laser Doppler (model P10, Moor Instruments, Wilmington, DE) was used to monitor the CBF for 5 min, both before and after MCAO, as well as during reperfusion for 5 min. Relative CBF post reperfusion had to rise to at least 50% of pre-ischemic levels in order for mice to be included for further analyses. During surgery procedures, body temperature was maintained by an electric warming blanket.

### Drug Administration

A selective NLRP3 inflammasome inhibitor MCC950 was given to mice at a dose of 10 mg/kg by intraperitoneal injection at indicated time points at the beginning of CS exposure. Mice that received an equal volume of vehicle (phosphate-buffered saline) were used as controls. Bindarit (Selleckchem, Houston, TX), an inhibitor of monocyte chemotactic protein synthesis, was diluted in 0.5% carboxymethylcellulose aqueous solution and administered at a dose of 50 mg/kg by oral gavage twice daily. Bindarit treatment was initiated on the same day as smoking and continued until the end of experiments. Control animals received an equal volume of carboxymethylcellulose (15–17).

### 2,3,5-Triphenyltetrazolium Chloride (TTC) Staining

TTC staining was used to evaluate infarct volume in our study. At days 1 and 3 after MCAO and reperfusion, mouse whole brains were obtained. After exposure for 5 min at −20°C, frozen whole brains were cut into 1 mm thick coronal slices starting at 1 mm from the frontal tips for TTC staining. A 2% (v/v) TTC solution (Sigma, St. Louis, MO, USA) was used to stain brain sections. Infarct areas were determined as absence of TTC stains and were quantified by Image Pro Plus analysis as previously described (13, 18).

### Neurological Function Assessment

At days 1 or 3 after MCAO, the modified Neurological Severity Score (mNSS) was adopted to evaluate neurodeficits. The scoring system comprises a set of terms to assess motor function (muscle and abnormal movement), sensory function (visual, tactile, and proprioceptive), and reflexes (pinna, corneal, and startle reflexes). The range of scores for mNSS is from 0 to 18. The rating scale defined as: a score of 13–18 indicates severe injury, 7–12 indicates moderate injury, and 1–6 indicates mild injury. Mice received a point if they failed to perform a task. Neurological deficit assessment was performed by investigators blinded to treatment groups, as described previously (13, 14, 19).

### Flow Cytometry

Single cell suspensions were prepared from brain, spleen, and peripheral blood and stained with fluorochrome-conjugated antibodies, as previously described (13, 14, 20). The antibodies used were: CD3 (145-2C11, 553066, BD Biosciences, San Jose, CA), CD4 (RM4-5, 552775, BD Biosciences, San Jose, CA), CD8 (53-6.7, 557654, BD Biosciences, San Jose, CA), CD11b (M1/70, 25-0112-82, eBioscience, San Diego, CA), CD19 (1D3, 152410, Biolegend, San Diego, CA), CD45 (30-F11, 12-0451-83, eBioscience, San Diego, CA), LY6C (BM8, 123119, Biolegend, San Diego, CA), Ly6G (1A8, 127614, Biolegend, San Diego, CA), and anti-NLRP3 antibody (Ab4207, Abcam, Cambridge, MA, USA); Alexa Fluor®488-conjugated donkey anti-goat IgG (H+L) was the secondary antibody (Invitrogen, Carlsbad, CA, USA). Fluorescence minus one (FMO) controls were stained at the same time. Flow cytometry was performed on a FACSAria flow cytometer. Data were analyzed using Flow Jo 7.6.1 software.

### Real-Time PCR

At day 1 after MCAO, total mRNA was extracted from the ischemic hemisphere brain tissue using a Trizol reagent (Invitrogen, Carlsbad, CA, USA) according to the manual instructions. One microgram of mRNA was reverse transcribed into cDNA using a PrimeScript^TM^ RT reagent Kit (TaKaRa, Shiga, Japan). An SYBR gene polymerase chain reaction (PCR) Master Mix (Roche, Indianapolis, IN, USA) was used to amplify the targeted gene sequence on the Opticon 2 Real-Time PCR Detection System (Bio-Rad). The primers used in our study are listed as follows: IL-1β forward: TGCCACCTTTTGACAGTGATG, IL-1β reverse: TGATGTGCTGCTGCGAGATT; IL-6 forward: GCTGGTGACAACCACGGCCT, IL-6 reverse: AGCCTCCGACTTGTGAAGTGGT; TNF-α forward: TATGGCTCAGGGTCCAACTC, TNF-α reverse: GGAAAGCCCATTTGAGTCCT; IL-10 forward: AAATAAGAGCAAGGCAGTGG, IL-10 reverse: GTCCAGCAGACTAAATACACAC; TGF-β forward: TGCGCTTGCAGAGATTAAAA, TGF-β reverse: CGTCAAAAGACAGCCACTCA; CCL2 forward: CTGCTGTTCACAGTTGCCG, CCL2 reverse: GCACAGACCTCTCTCTTGAGC; β-actin forward: CACCCGCGAGTACAACCTTC, β-actin reverse: CCCATACCCACCATCACACC; β-actin served as a reference gene.

### Statistical Analysis

Animals were randomly assigned to treatment conditions. Results were analyzed by investigators blinded to the treatment. Data are shown as mean ± SD. Statistical analyses was performed using Graphpad 6.0 software. Statistical significance was determined by the two-tailed unpaired Student's *t*-test for two groups and one-way ANOVA followed by Tukey *post-hoc* test for three or more groups. Values of *p* < 0.05 were considered significant.

## Results

### Exposure to Cigarette Smoke Exacerbates Ischemic Brain Injury in Mice

**Figure 1:**
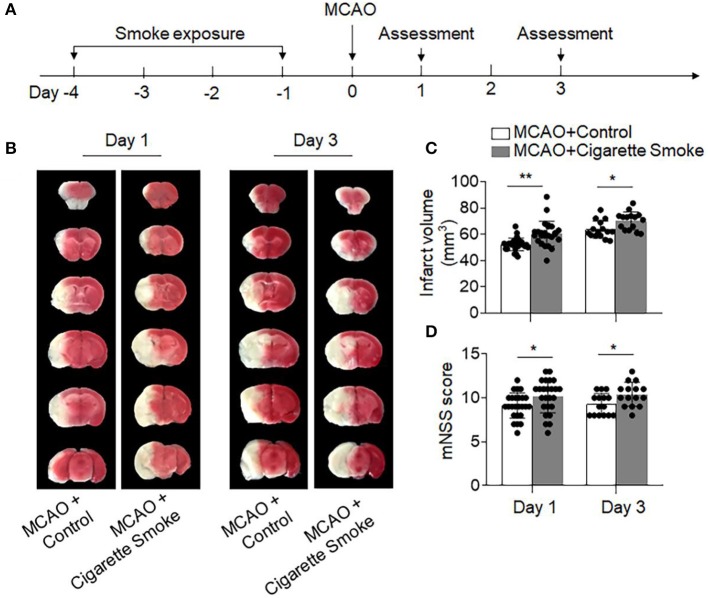
Exposure to cigarette smoke-augmented neurodeficits and brain infarction after ischemia. **(A)** Schematic diagram illustrates experimental design. C57BL/6 mice were exposed to cigarette smoke for 4 consecutive days. Mice receiving normal air were used as controls. MCAO was performed at day 0. At days 1 and 3 after MCAO and reperfusion, neurodeficits and brain infarct volume were assessed. **(B)** TTC-stained brain sections from mice receiving cigarette smoke or normal air at indicated time points after MCAO and reperfusion. Summarized bar graphs show **(C)** infarct volume and **(D)** modified Neurological Severity Score (mNSS) in mice receiving cigarette smoke or normal air at indicated time points after MCAO and reperfusion. *n* = 25 per group at day 1, *n* = 15 per group at day 3. Mean ± SD. ^*^P < 0.05, ^**^P < 0.01.

To assess the impact of cigarette smoke on ischemic brain injury, we measured neurodeficits and infarct volume in mice subjected to exposure of cigarette smoke followed by brain ischemia induced by 60 min transient MCAO. Mice were exposed to cigarette smoke twice daily for 4 days prior to MCAO, and MCAO was induced at 24 h after the last exposure (Figure 1A). Neurodeficits and infarct volume were assessed at days 1 and 3 after MCAO and reperfusion. We found that exposure to cigarette smoke significantly aggravated neurodeficits and infarct volume after MCAO, as compared to controls inhaling normal air (Figures 1B–D). The infarct volumes of these two groups were as follows: Day 1: [MCAO + Control: 52.31 ± 4.85 vs. MCAO + Cigarette Smoke: 60.53 ± 9.46, mm^3^, *p* = 0.0003], Day 3: [MCAO + Control: 63.69 ± 6.58 vs. MCAO + Cigarette Smoke: 70.32 ± 6.83, mm^3^, *p* = 0.01].

To measure the amount of cigarette smoke taken by the mice, we measured the levels of cotinine in plasma. As the primary metabolite of nicotine, cotinine is used as a biomarker for smokers due to its longer half-life when compared to nicotine (12). After the last treatment of cigarette smoke, the average cotinine level was 423.5 ng/ml (Supplementary Figure 1A), which is within the range of 10–500 ng/ml in plasma from active smokers (21, 22). In addition, cigarette smoke did not affect CBF (Supplementary Figure 1B), blood gas parameters, or physiological variables (Supplementary Table 1). Together, these results indicated that exposure to cigarette smoke similar to active smokers is sufficient to exacerbate ischemic brain injury.

### Exposure to Cigarette Smoke Enhances Brain Inflammatory Milieu After Brain Ischemia

**Figure 2:**
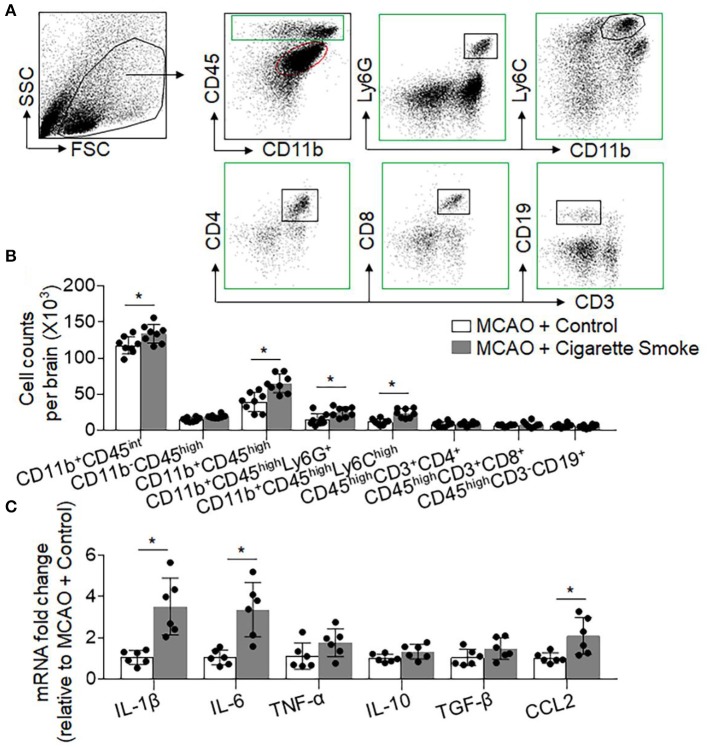
Cigarette smoke-augmented brain infiltration of leukocytes and production of pro-inflammatory factors in MCAO mice. At day 3 after MCAO and reperfusion, brain tissue was harvested from mice receiving cigarette smoke or normal air. Thereafter, single cell suspensions or brain homogenates were prepared from these tissues. **(A)** Gating strategy for flow cytometry analysis of CD11b^+^CD45^int^ , CD11b^−^CD45^high^, CD11b^+^CD45^high^, CD45^high^CD11b^+^LY6G^+^, CD45^high^CD11b^+^LY6C^high^, CD45^high^CD3^+^CD4^+^, CD45^high^CD3^+^CD8^+^, and CD45^high^CD3^−^CD19^+^ cells. **(B)** Bar graph shows summarized counts of indicated cell subsets. *n* = 8 per group. Mean ± SD. ^*^P < 0.05. **(C)** Bar graph shows results from mRNA expression levels of cytokines and chemokines in homogenates of the ipsilateral hemisphere brain tissues from indicated groups of mice. *n* = 6 per group. Mean ± SD, ^*^P < 0.05, vs. control group.

We sought to understand the influences of smoke exposure on brain inflammation after brain ischemia. Using flow cytometry, we examined the counts of brain-infiltrating leukocytes and microglia at day 3 after MCAO (Figure 2A). The counts of brain-infiltrating leukocytes (CD45^high^), neutrophils (CD45^high^CD11b^+^Ly6G^+^), monocytes (CD45^high^CD11b^+^LY6C^high^), and microglia (CD11b^+^CD45^int^) were significantly increased in MCAO mice receiving cigarette smoke exposure (Figure 2B). In addition, the upregulation of pro-inflammatory factors, such as IL-6, IL-1β, and CCL2, was also seen in mice receiving cigarette smoke exposure at day 1 after MCAO (Figure 2C). These results suggest that cigarette smoke exposure augments post-ischemic brain inflammation.

### Smoke Exposure Mobilizes Neutrophils and Monocytes in the Periphery

**Figure 3:**
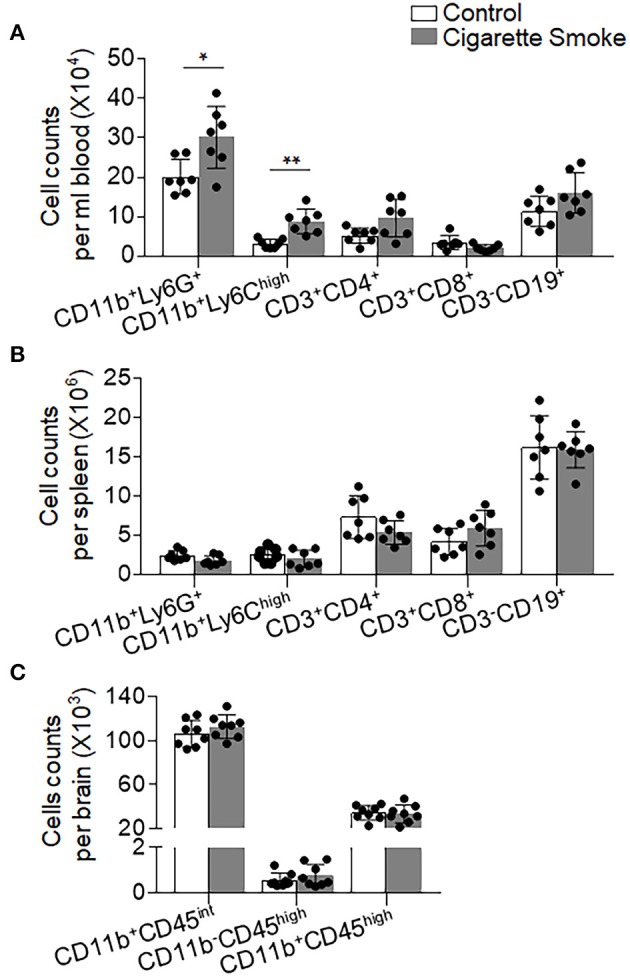
Cigarette smoke alters neutrophil and monocyte responses in the periphery. Blood, spleen, and brain samples were harvested from groups of mice receiving 4 consecutive days of exposure to cigarette smoke or normal air. Bar graphs shows counts of CD11b^+^LY6G^+^, CD11b^+^LY6C^high^, CD3^+^CD4^+^, CD3^+^CD8^+^, and CD3^−^CD19^+^ cells in the **(A)** blood and **(B)** spleen of indicated groups of mice. **(C)** Bar graphs show counts of CD11b^+^CD45^int^, CD11b^−^CD45^high^ , and CD11b^+^CD45^high^ cells in the brains of indicated groups of mice. *n* = 7 per group. Mean ± SD. ^*^P < 0.05, ^**^P < 0.01.

As smoke exposure has been linked to elevation of circulating leukocytes (23–25) and our findings show an increase of brain-infiltrating neutrophils and monocytes in MCAO mice receiving smoke exposure, we sought to determine the influence of cigarette smoke on immune responses prior to the induction of brain ischemia. For this purpose, we measured cell counts of immune cell subsets including neutrophils, monocytes, and lymphocytes in the spleen and blood of mice receiving cigarette exposure without MCAO. We found that the counts of circulating neutrophils (CD11b^+^Ly6G^+^) and pro-inflammatory monocytes (CD11b^+^Ly6C^high^) were significantly increased in mice receiving cigarette exposure (Figure 3A). In contrast, the counts of other immune cell subsets including CD4^+^ T cells (CD3^+^CD4^+^), CD8^+^ T cells (CD3^+^CD8^+^), and B cells (CD3^−^CD19^+^) did not have significant changes (Figure 3A). Similar numbers of these immune cell subsets were seen in the spleen of mice receiving exposure to cigarette smoke vs. normal air (Figure 3B). Of note, cigarette smoke alone did not affect the numbers of microglia and infiltrating leukocytes in the brain (Figure 3C). These results suggest that exposure to cigarette smoke is sufficient to induce mobilization of peripheral neutrophils and pro-inflammatory monocytes.

### Inhibition of Neutrophil and Monocyte Recruitment Alleviated Cigarette Smoke-Induced Exacerbation of Ischemic Brain Injury

**Figure 4:**
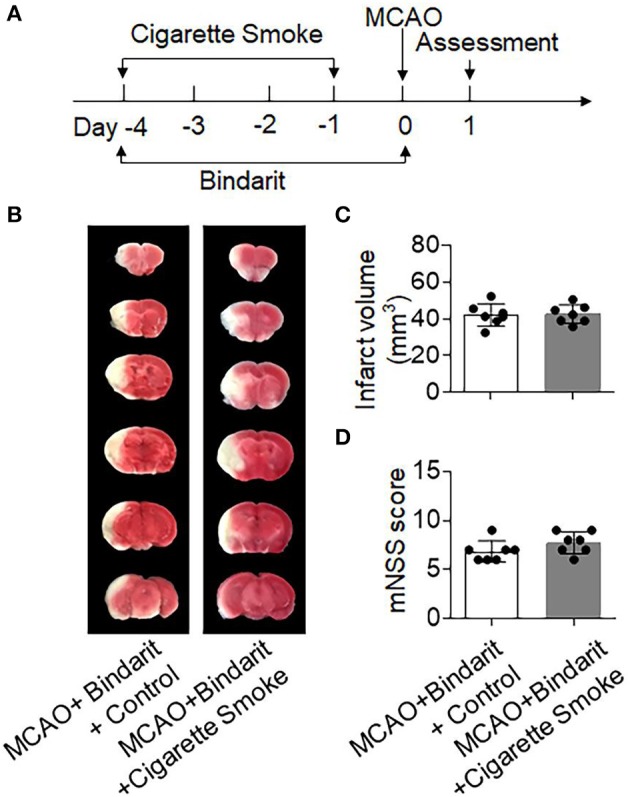
Inhibition of neutrophil and monocyte recruitment mitigated cigarette smoke-induced exacerbation of ischemic brain injury. **(A)** Schematic diagram illustrates drug administration and experimental design. Mice received Bindarit (50 mg/kg, orally twice daily) at the indicated time points. MCAO mice receiving an equal volume of vehicle were used as controls. At day 1 after MCAO, neurodeficits and brain infarct volume were assessed. **(B)** TTC-stained brain sections from indicated groups of mice receiving exposure to cigarette smoke or normal air at 24 h after MCAO and reperfusion. **(C)** Bar graph shows brain infarct volume in indicated groups of mice. **(D)** Summarized mNSS in indicated groups of mice. *n* = 7 mice per group. Mean ± SD.

Next, we sought to understand whether mobilization of neutrophils and monocytes after cigarette smoke contributes to exacerbation of stroke severity. As an inhibitor of MCP-1/CCL2 synthesis, bindarit has been shown to effectively block chemokine-driven recruitment of monocytes and neutrophils into the ischemic brain (17, 26–28). Indeed, we found that bindarit treatment significantly decreased neutrophil (CD45^high^CD11b^+^Ly6G^+^) and monocyte (CD45^high^CD11b^+^Ly6C^high^) infiltration into the brain and attenuated ischemic brain injury after MCAO (Supplementary Figures 2A–C). Importantly, we observed that bindarit treatment was able to diminish cigarette smoke-induced aggravation of ischemic brain injury (Figures 4A–D). The infarct volumes of these two groups were as follows: [MCAO + Bindarit + Control: 42.12 ± 6.08 vs. MCAO + Bindarit + Cigarette Smoke: 42.51±5.05, mm^3^, *p* = 0.89]. These results suggest that mobilization of neutrophils and monocytes may contribute to augmented stroke severity after cigarette smoke.

### The Aggravation of Cigarette Smoke on Brain Injury Is Dependent on NLRP3 Pathway

**Figure 5:**
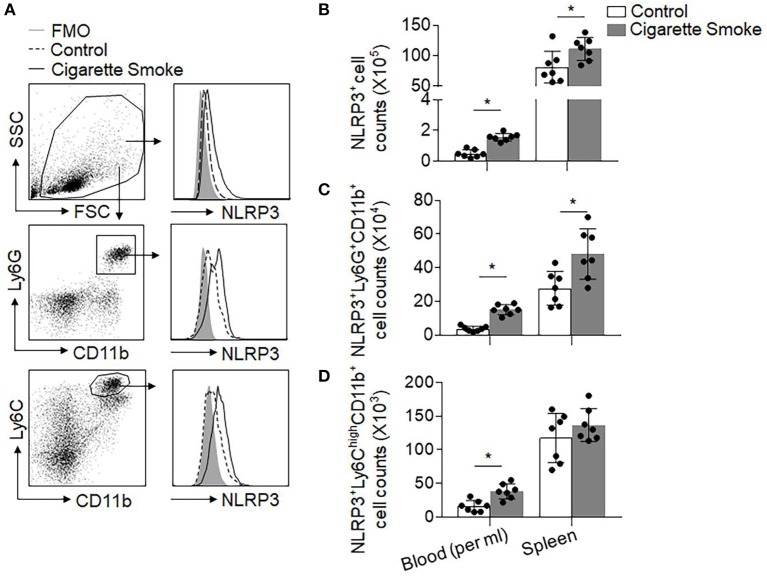
Cigarette smoke induces upregulation of NLRP3 inflammasome in peripheral neutrophils and monocytes. Blood and spleen samples were harvested from groups of mice receiving 4 consecutive days of exposure to cigarette smoke or normal air. **(A)** Gating strategy for CD11b^+^Ly6G^+^ neutrophils and CD11b^+^Ly6C^high^ monocytes expressing NLRP3. FMO, fluorescence minus one. **(B–D)** Quantification of cell counts of the indicated immune cell populations per ml of blood or whole spleen in groups of mice receiving exposure to cigarette smoke or normal air. *n* = 7 per group. Mean ± SD. ^*^P < 0.05.

The NOD-like receptor (NLR) pyrin domain–containing protein 3 (NLRP3) inflammasome is a key effector protein of neutrophils and monocytes that boosts local inflammation and contributes to ischemic brain injury (15, 29). Therefore, we postulated that NLRP3 inflammasome activation contributes to cigarette smoke-induced exacerbation of stroke injury. Intriguingly, we found that cigarette smoke induced a dramatic increase of NLRP3^+^ neutrophils (CD11b^+^Ly6G^+^) or monocytes (CD11b^+^Ly6C^high^) in the blood and spleen prior to MCAO (Figures 5A–D). These results indicate augmented NLRP3 inflammasome activity in peripheral neutrophils and monocytes after exposure to cigarette smoke, suggesting smoke priming of neutrophil and monocyte responses.

**Figure 6:**
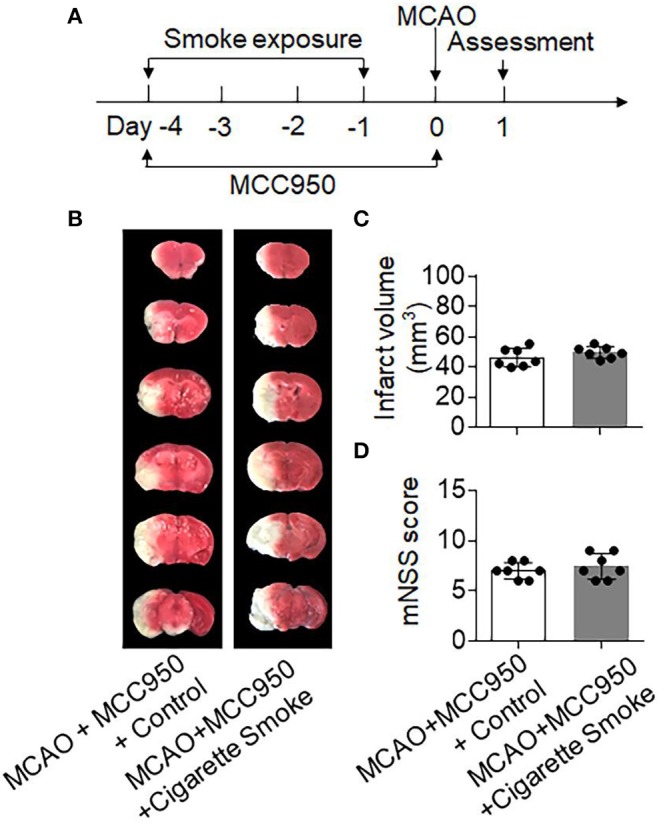
Inhibition of NLRP3 inflammasome mitigated cigarette smoke-induced exacerbation of ischemic brain injury. **(A)** Schematic diagram illustrates drug administration and experimental design. Mice received MCC950 (10 mg/kg, i.p.) at indicated time points. Mice receiving an equal volume of vehicle were used as controls. At day 1 after MCAO, neurodeficits and brain infarct volume were assessed. **(B)** TTC-stained brain sections from indicated groups of mice receiving exposure to cigarette smoke or normal air at 24 h after MCAO and reperfusion. **(C)** Bar graph shows brain infarct volume in indicated groups of mice. **(D)** Summarized mNSS in indicated groups of mice. *n* = 7 mice per group. Mean ± SD.

In the brain, we found that prior to MCAO there were few NLRP3^+^ neutrophils and monocytes that infiltrated the brain of both normal air- and cigarette smoke-treated mice. Upon MCAO ictus, we observed more NLRP3^+^ neutrophils and monocytes in the brain from mice exposed to cigarette smoke as compared to the control mice (Supplementary Figures 3A,B). To determine the contribution of NLRP3 inflammasome activity to cigarette smoke-induced stroke exacerbation, we used a selective NLRP3 inflammasome inhibitor MCC950 (15, 30). We found that MCC950 was able to reduce neutrophils and monocytes that expressed NLRP3 in the brain and spleen after ischemia (Supplementary Figures 4A,B). Intriguingly, the detrimental effects of cigarette smoke on ischemic brain injury was diminished in MCAO mice receiving MCC950 (Figures 6A–D). The infarct volumes of these two groups were as follows: [MCAO + MCC950 + Control: 46.32 ± 6.12 vs. MCAO + MCC950 + Cigarette Smoke: 49.67 ± 3.91, mm^3^, *p* = 0.25]. All in all, these results indicate that cigarette smoke-induced stroke exacerbation involves NLRP3 inflammasome activity.

## Discussion

This study provides the first definite evidence that exposure to cigarette smoking exacerbates post-ischemic brain injury and inflammation. We document here that cigarette smoke mobilizes peripheral neutrophils and monocytes that contribute to exacerbated stroke severity. In addition, we report that the deleterious effects of cigarette smoke involve NLRP3 inflammasome activity in neutrophils and monocytes. This study thus provides novel evidence supporting the notion that cigarette smoke primes peripheral innate immune responses that are detrimental to ischemic stroke.

The finding of augmented neurodeficits and infarct volume in mice inhaling cigarette smoke suggests that cigarette smoke alone is sufficient to exacerbate stroke severity. Although under different experimental conditions, this result is consistent with previous studies showing that nicotine, a component of cigarette smoke, aggravates brain injury in experimental stroke models (9, 31). It's notable that the influences of cigarette smoke on stroke severity may be affected by the length of exposure duration and exposure dose. A recent study reported that cigarette smoke exposure at a dose of nine cigarettes per day had no significant impact on brain infarction or neurological deficits in mice subjected to 30–40 min MCAO (32). The reasons that caused this discrepancy may be at least partially caused by the different exposure protocol of cigarette smoke and ischemia duration. Because such factors could impact cigarette smoke priming of peripheral immune cells, this likely leads to different baseline inflammation prior to ischemia.

Consistent with previous findings of elevated circulating neutrophils and monocytes in active smokers (23, 27, 28), we found that cigarette smoke leads to increased mobilization of peripheral neutrophils and monocytes. In addition, these changes were restricted to neutrophils and monocytes but not to other tested immune cell subsets. Together, these findings support the notion that cigarette smoke induces priming of peripheral neutrophils and monocytes that predispose the host to ischemic brain injury. In support of this view, we found that inhibition of neutrophil and monocyte recruitment abolished the exacerbated stroke severity after smoke exposure. It's also noteworthy that the number of microglia and brain-infiltrating leukocytes were not affected by cigarette smoke before MCAO, suggesting that smoke-induced priming of immune responses mainly occurs in the peripheral compartment rather than the brain, at least in our experimental setting.

The NLRP3 inflammasome is a key effector protein in myeloid cells such as neutrophils and monocytes that amplifies inflammatory responses and ischemic brain injury by facilitation of caspase-1 and interleukin (IL)−1 β processing (15, 29). Of interest, we found that cigarette smoke upregulated NLRP3 inflammasome in peripheral neutrophils and monocytes. In addition, NLRP3 inflammasome activity contributed to cigarette smoke-induced exacerbation of stroke severity. These results provide novel evidence regarding smoke priming of neutrophil and monocyte responses, although the precise operating mechanisms through which cigarette smoke upregulates inflammasome activity remain uncertain and require future investigation.

There are also limitations to this study. First, our experimental dose of cigarette smoke exposure in this study is similar to heavy smokers (cotinine levels above 300 ng/mL are seen in heavy smokers). Therefore, it would be interesting to study the impact of chronic low dose smoke exposure on the systemic immune response and ischemic brain injury. Second, as cigarette smoke contains more than 4,000 components, a control group exposed to other sources of smoke may facilitate result interpretation. Although other smoke inhalation such as that of carbon monoxide may also induce an immune response in the lungs, caution may be needed before generalizing our findings to include all smoke inhalation conditions such as carbon monoxide, because previous studies also reported that carbon monoxide may reduce circulating leukocyte numbers and activity (33, 34). Third, amounting evidence suggests that targeting inflammation and immune responses is a viable approach to rescuing brain tissue and improving outcomes after stroke (2, 35). However, translation to human medicine has been disappointing. The reasons might be that targeting the highly dynamic events that occur during inflammation in the relatively inaccessible brain microenvironment is challenging, and an incomplete understanding of the interactions between the immune system and the brain during stroke limits progress. Therefore, more caution should be used when interpreting the translational potential of our findings.

## Conclusions

Our findings suggest that cigarette smoke induces priming of peripheral neutrophils and monocytes that contribute to aggravated ischemic brain injury, implying that the restriction of exposure to cigarette smoke would reduce the detrimental consequences of ischemic stroke.

## Data Availability Statement

All datasets generated for this study are included in the article/Supplementary Material.

## Ethics Statement

All animal experiments were approved by the Institutional Animal Care and Use Committees of Tianjin Medical University General Hospital (Tianjin, China). This study was conducted in accordance with the National Institutes of Health Guide for the Care and Use of Laboratory animal in China.

## Author Contributions

HL, XL, SG, DW, and XG acquired and analyzed the data. ML, HL, YL, XW, ZC, and HM interpreted the results. HM, QL, and ML formulated the study concept. ML designed the study, and drafted the manuscript. YL and ML acquired funding for this study.

### Conflict of Interest

The authors declare that the research was conducted in the absence of any commercial or financial relationships that could be construed as a potential conflict of interest.
