# Supplementary material for: Exposure to Cigarette Smoke Augments Post-ischemic Brain Injury and Inflammation via Mobilization of Neutrophils and Monocytes
Source: Front Immunol. 2019 Nov 7;10:2576. doi: 10.3389/fimmu.2019.02576 (PMC6853894; doi:10.3389/fimmu.2019.02576)
Supplement: Supplementary file 1 [file Data_Sheet_1.pdf]

Supplemental Materials

Table 1. Blood gas parameters and physiological variables.

	Control	Cigarette smoke
pH units	7.35±0.09	7.42±0.02
PCO_2, mm Hg	37.5±2.2	32.3±2.1
PO_2, mm Hg	101.7±1.8	102.8±3.4
K+, mmol/l	4.11±0.2	4.03±0.1
Glucose, mmol/l	12.4±1.1	12.3±0.6
Rectal temperature, °C	36.8±0.2	37.0±0.2

Blood gas parameters and physiological variables were measured in mice receiving 4 days exposure to cigarette smoke or normal air. n=8 per group. Mean ± SD.

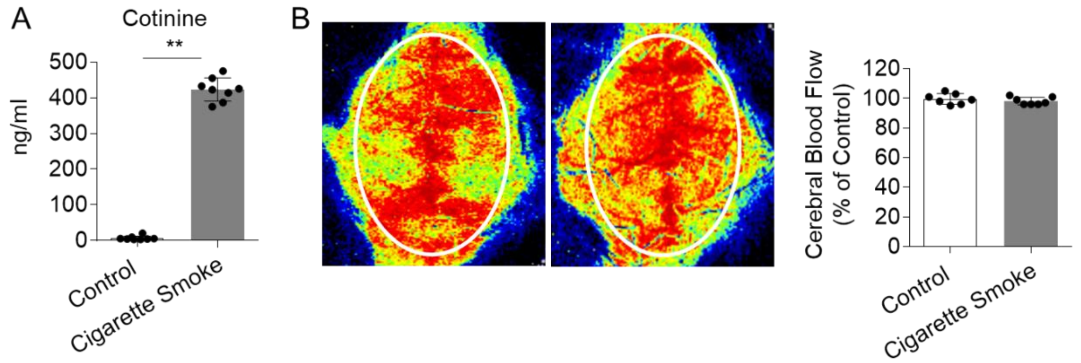

Supplementary Figure 1. Cotinine levels and cerebral blood flow after smoke exposure.

A. Circulating cotinine levels were measured in mice receiving 4 days exposure to cigarette smoke or normal air. n=8 per group. Mean ± SD. **P < 0.01. **B**. **Left**: images of cerebral blood flow (CBF) before MCAO surgery in normal air exposure and smoke exposure group. **Right**: Quantification of CBF. Results show normalized cerebral blood flow. n=7 mice per group. Mean ± SD.

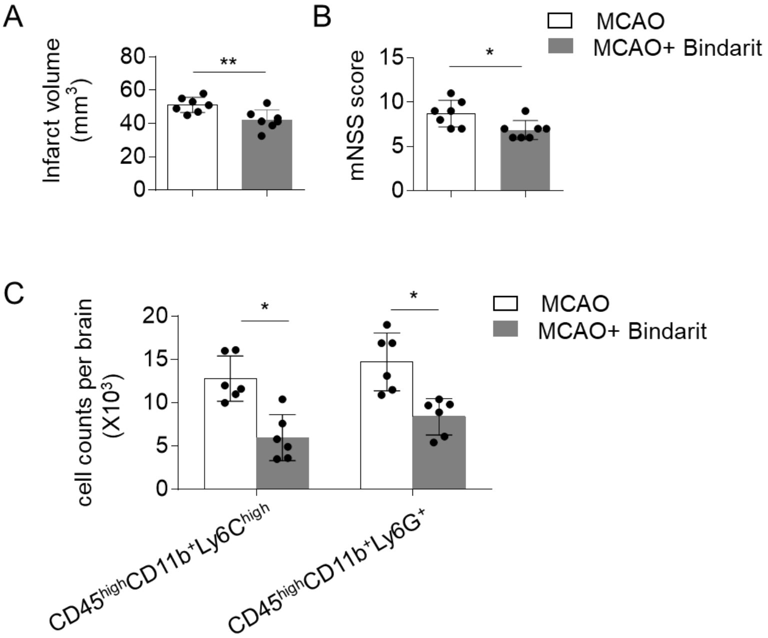

Supplementary Figure 2. Bindarit treatment reduced neurodeficits and infarct volume after MCAO. Mice received 4 consecutive days of bindarit (50 mg/kg, orally twice daily) or equal volume of vehicle before MCAO. At day 1 post-MCAO, neurodeficits and brain infarct volume were assessed. **A-B**. Summarized bar graph shows infarct volume and modified Neurological Severity Score (mNSS) of mice receiving bindarit or vehicle. n = 7 per group. *P<0.05, **P< 0.01. **C**. Quantification of cell counts of neutrophils ($CD45^{high}CD11b^{+}Ly6G^{+}$) and pro-inflammatory monocytes ($CD45^{high}CD11b^{+}Ly6C^{high}$) in the brain of mice receiving bindarit or vehicle. n=6 per group. *P < 0.05. Mean ± SD.

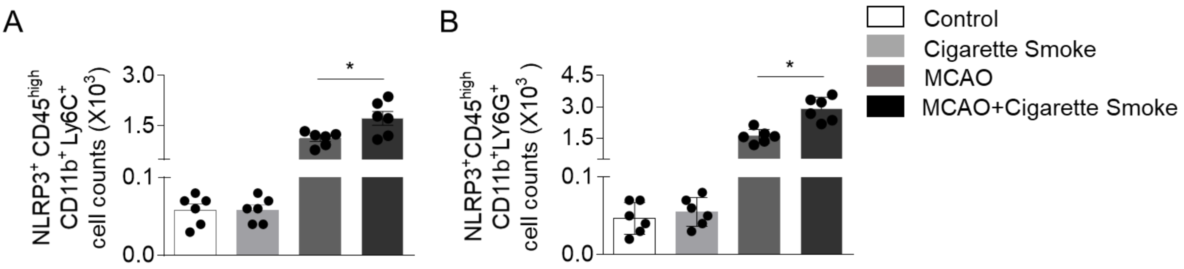

Supplementary Figure 3. Cigarette smoke increased cell counts of brain-infiltrating neutrophils and monocytes that express NLRP3 after MCAO. Brain samples were harvested for flow cytometry analysis from groups of mice receiving 4 consecutive days for exposure to cigarette smoke or normal air prior to

MCAO surgery or post MCAO surgery under 4 days cigarette exposure. **A-B**. Bar graph shows the quantification of cell counts of NLRP3$^+$ monocytes (CD45^{high}CD11b$^+$Ly6C$^+$) and NLRP3$^+$ neutrophils (CD45^{high} CD11b$^+$Ly6G$^+$) in the brain tissue at day1 after MCAO. n=6 per group. *P < 0.05, Mean ± SD.

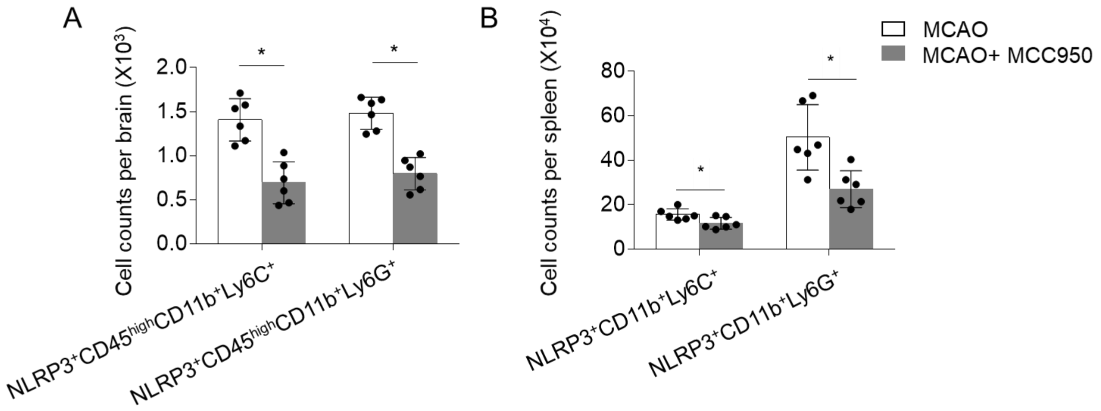

Supplementary Figure 4. MCC950 treatment decreased neutrophil and monocytes that express NLRP3 in the brain and spleen after MCAO. Mice was treated with 4 consecutive days of MCC950 (10 mg/kg, i.p.) or equal volume of vehicle before MCAO. At day 1 after MCAO, brain and spleen samples were harvested for flow cytometry analysis. **A-B**. Quantification of cell counts of neutrophils (CD45^{high} CD11b$^+$Ly6G$^+$) and monocytes (CD45^{high}CD11b$^+$Ly6C$^+$) that express NLRP3 in the brain (A) and spleen (B). n=6 per group. *P < 0.05. Mean ± SD.
